# Healthcare Professionals’ Application and Integration of Physical Activity in Routine Practice with Older Adults: A Qualitative Study

**DOI:** 10.3390/ijerph182111222

**Published:** 2021-10-26

**Authors:** Conor Cunningham, Roger O’Sullivan

**Affiliations:** 1 Institute of Public Health, Belfast BT1 4JH, UK; roger.osullivan@publichealth.ie; 2 Bamford Centre for Mental Health and Wellbeing, Ulster University, Belfast BT37 0QB, UK

**Keywords:** physical activity, healthcare professionals, older adults, theoretical domains framework, policy, behaviour change

## Abstract

Healthcare professionals (HCPs) have a key role in promoting physical activity, particularly among populations at greatest risk of poor health due to physical inactivity. This research explored HCPs’ knowledge, decision making, and routine practice of physical activity promotion with older adults. Furthermore, it aimed to enhance our understanding of the supports that HCPs need to effectively promote physical activity in routine practice across a wide range of healthcare professions, settings, and sectors. Semi-structured online interviews were completed with HCPs between November 2020–March 2021. Data were first analysed by coding instances within the transcripts, mapping onto relevant Theoretical Domains Framework (TDF) domains utilising a deductive thematic analysis approach. The data were then analysed utilising an inductive approach to thematically generate explanatory subthemes within the identified domains. Participants (*n* = 63) included general practitioners (15.87%), occupational therapists (30.16%), physiotherapists (38.10%), and nurses (15.87%) from the island of Ireland (Ireland and Northern Ireland). Of those interviewed, 10 (15.87%) were male and 53 (84.13%) were female. Two thirds (65.08%) were HCPs practicing in Ireland. Domains and subthemes related to the application of physical activity, and emergent themes on developing practice to support the application and integration of physical activity in routine practice are discussed. HCPs identified that focused education, appropriate training, and access to tailored resources are all essential to support the promotion of physical activity in routine practice. For such supports to be effective, a ‘cultural shift’ is required in HCP training and health service provision to adopt the growing evidence base that physical activity promotion must be part of disease prevention and treatment in routine practice. HCPs highlighted a range of areas for service development to support them to promote physical activity. Further research is required to explore the feasibility of implementing these recommendations in routine practice.

## 1. Introduction

International guidelines recommend that all older adults (65+ years) should aim to do at least 150 min of moderate intensity or 75 min of vigorous intensity aerobic activity throughout the week, with muscle strengthening and multicomponent balance training on 2 or more days per week [1]. For older adults meeting international physical activity recommendations, there is a significant reduction in the risk of all-cause mortality, Alzheimer’s disease, and incident depression [2]. In addition to recommending that all older adults should undertake regular physical activity, these guidelines also emphasise the benefits of even small increases in physical activity and less time spent sedentary throughout the day, prompting the “move more, sit less” message [1]. However, for many, ageing is defined by rapid declines in levels of physical activity, loss of mobility and functional independence, and premature morbidity [3]. This stage of life therefore represents an important period to promote physical activity to improve functions of daily living and slow progression of disease and disability [4].

Physical activity promotion has increasingly been recognised as a priority for public health supported by the development of policies and interventions [4]. These actions recognise that addressing inactivity requires a ‘whole of society’ approach, with action across different sectors [5]. Key strategic objectives of this ‘systems-based’ approach in the health sector are to implement and strengthen systems of patient assessment and counselling on increasing physical activity and reducing sedentary behaviour by appropriately trained healthcare professionals (HCPs) [5] and to enhance the provision of, and opportunities for, appropriately tailored programmes and services aimed at increasing physical activity in older adults [6].

HCPs are well placed to promote physical activity through both structured and opportunistic contact [7,8]. They serve as influential sources of information and guidance with a wide range and number of patients and frequently engage with those in most need of physical activity advice, e.g., people with type 2 diabetes, depression, joint and back pain [2,9]. Integrating physical activity counselling and referral as part of routine brief interventions in primary healthcare systems is identified as a ‘best buy’ in public health with proven effectiveness in decreasing the burden of non-communicable diseases and improving quality of life [2,10].

While the promotion of physical activity should be a core competence for all primary healthcare professionals [11], challenges exist, such as time constraints, perceived lack of patient engagement, and competing priorities [12]. In addition, there is a recognition of the need for greater teaching in this area at both undergraduate and postgraduate levels [12], and for the provision of additional in-service training and evidence-informed resources to support effective promotion of physical activity in routine practice [13]. Our own research in this area identified a need for continuing professional education and skill development [14], and while programmes have been introduced to develop knowledge of how to carry out a brief intervention with patients or service users, there is still a broad recognition of the need for further supports in this area to enable HCPs to effectively assess, counsel, and support their patients to increase levels of physical activity [14,15,16].

This research sought to develop our understanding of the supports (individual and structural/organisational) that HCPs need to effectively promote physical activity (through assessment, discussion, and prescription) in routine practice with older adults. Furthermore, this research aimed to enhance our understanding of knowledge (how it is developed and implemented in practice) and decision making (barriers/facilitators and models of good practice in physical activity promotion) in relation to physical activity promotion as part of routine care with older adults on the island of Ireland (Ireland and Northern Ireland).

## 2. Materials and Methods

This qualitative study was the third phase of a broad programme of research comprising a systematic review of reviews, a quantitative survey, and this qualitative follow- up. The systematic review of reviews (Phase 1) provided a comprehensive and systematic overview of epidemiological evidence from previously conducted research to assess the associations of physical activity with physical and mental health outcomes in older adults [2]. Phase 2 involved a cross-sectional survey of HCPs’ knowledge and routine practice of physical activity promotion with older adults on the island of Ireland (Ireland and Northern Ireland) [14]. The purpose of this qualitative component (Phase 3) was to build on the survey findings to further enhance our understanding of HCPs’ knowledge and decision making in relation to physical activity promotion and explore HCPs’ own views on the supports that are needed to effectively apply and integrate physical activity promotion in routine practice across a wide range of healthcare professions, settings, and sectors.

### 2.1. Design and Sampling

This qualitative component used semi-structured, online interviews. Respondents (general practitioners, occupational therapists, nurses, and physiotherapists) from the previous survey (Phase 2) were asked if they consented to future contact from the research team [14]. Those who agreed were e-mailed an invitation to participate (including participant information sheet and consent form). Other participants were recruited through professional bodies for general practice, physiotherapy, nursing, and occupational therapy on the island of Ireland. A purposive sampling method was used to ensure that each profession was represented. Sampling continued until there was a consensus by the authors that theoretical and content saturation had occurred. A total of *n* = 63 HCPs participated (see Table 1). The study was conducted according to the guidelines of the Declaration of Helsinki and independently peer reviewed.

### 2.2. Data Collection

An interview schedule was developed and pilot tested by the research team based on the key theoretical domains explored in the survey which identified, in particular, the domains of Knowledge, Skills and Behavioural Regulation [14]. The schedule included questions on key topics related to national guidelines for physical activity for older adults; knowledge and practice of assessment/discussion/prescription of physical activity with patients as part of routine care; perceived opportunities to promote physical activity in day-to-day practice; the supports (individual and structural/organisational) that HCPs need to effectively promote physical activity (through assessment, discussion, and prescription) in routine practice, and questions related to implications of the COVID-19 pandemic on routine practice and views on the role of physical activity promotion to older adults in light of the pandemic. Analysis and findings of interview data related to the COVID-19 pandemic are reported in a separate publication.

Semi-structured interviews, lasting 30–45 min, were conducted by C.C. (who is experienced in qualitative methods), using an encrypted online phone service (Zoom) between November 2020–March 2021. Written and verbal informed consent was obtained prior to commencing the interview. Interviews were digitally recorded and coded audio files were transcribed verbatim. Any identifiable information was removed from coded transcripts.

### 2.3. Data Analysis

Data were analysed using NVivo software (version 12.0 Plus, QSR International, Melbourne, Australia). To establish an understanding of the application and integration of physical activity promotion in routine practice for HCPs, the data were first analysed by coding instances within the transcripts, mapping onto relevant Theoretical Domains Framework (TDF) domains [17], utilising a deductive thematic analysis approach. The 14-domain TDF is an integrative framework of theories of behaviour change which has been used to identify influences on HCP behaviour in the implementation of evidence-based recommendations [17] by analysing the social, environmental, cognitive, and affective influences on HCP practice [18]. The data were then analysed by utilising an inductive approach to thematically generate explanatory subthemes within the identified domains. One researcher (C.C.) conducted the coding of all transcripts, mapping of sub-themes, and data synthesis. A second researcher (R.O.) independently analysed a random sample of the interviews (20%). Any differences were discussed, and a consensus reached to ensure appropriateness of coding and mapping. The STROBE Checklist was used to guide the reporting of items to be included in reports of cross-sectional studies [19].

## 3. Results

A total of 63 individuals participated in the interviews, at which point no new emerging themes were identified. Participants included general practitioners (15.87%), occupational therapists (30.16%), physiotherapists (38.10%), and nurses (15.87%) from the island of Ireland. Participant characteristics are presented in Table 1. Of those interviewed, 10 (15.87%) were male and 53 (84.13%) were female. The majority of those interviewed had 16–20 years of practice experience (31.75%, *n* = 20). The proportion of those interviewed who reported that they worked in the public sector was 87.3% (*n* = 55) and two thirds (65.08%) were HCPs practicing in Ireland.

A summary of domains and subthemes related to knowledge of physical activity and application to routine practice, and area(s) for potential knowledge/practice development are presented in Table 2. Emergent themes on developing practice to support the application and integration of physical activity promotion are presented in Table 3. Additional HCP quotations coded during data analysis are presented in Supplementary Table S1.

In this section, we provide an overview of the emergent domains and subthemes from the analysis of interview data grouped under ‘applying’ and ‘integrating’ physical activity promotion to routine practice.

### 3.1. Applying Physical Activity to Routine Practice

At the beginning of each interview, HCPs were asked about their knowledge of physical activity guidelines in their jurisdiction, how and where they got their knowledge of physical activity and health, and how they felt this knowledge applied to routine practice.

### 3.2. TDF Domain: Knowledge

#### 3.2.1. Emergent Subtheme: Knowledge and Understanding of the Benefits of Physical Activity for Patients’ Health

There was a broad recognition of the benefits of physical activity for patients’ health, across a range of both physical and mental health conditions that present in routine practice, and this was a consistent subtheme irrespective of healthcare setting, whether that be in the acute setting, in the community setting, or in residential care.

#### 3.2.2. Emergent Subtheme: Source(s) of Knowledge Development

Several participants referred to current programmes designed to develop knowledge and application of brief interventions in routine practice (e.g., Making Every Contact Count), and more broadly, participants reported a wide range of sources to support their knowledge development. These represented: online resources, conferences, seminars, and webinars; professional bodies, societies, faculties, and associations; professional networks, and special interest groups (see Table 2).

#### 3.2.3. Emergent Subtheme: Initial and Continuing Professional Education

Many HCPs acknowledged that their fundamental awareness of the role of physical activity in prevention and treatment of disease did not come from their undergraduate/initial education and training, and more specifically, that there is a need for continuing professional development (CPD) in relation to promoting physical activity for older adults’ health. Many HCPs discussed how an interest in physical activity in general, and in the role that physical activity plays in health more specifically, had helped to define their continuing knowledge development.

#### 3.2.4. Emergent Subtheme: Knowledge of Physical Activity Guidelines

Knowledge of physical activity guidelines varied considerably amongst HCPs. Some reported that they were aware of but could not recall specific components of the guidelines. Others reported that they utilise the guidelines daily and integrate them into every patient consultation (where appropriate). Most HCPs identified that using guidelines for physical activity in routine practice required a tailored approach, which followed initial discussions with a patient about their levels of physical activity or was contingent on their professional judgement and decision making in relation to how physical activity could be assessed, discussed, and prescribed with each patient.

### 3.3. TDF Domain: Belief about Consequences

Several HCPs cited their belief in the benefits of physical activity and exercise for both patients’ health and their own health. For example,

‘*…but I suppose we believe, I think that’s the thing, I have no doubt of the benefits of exercise. And I even say it myself, even going back to my student days, I can remember people saying that they studied better when they were physically fitter. And I think there’s… so I think the whole mind and body thing, it helps both, is very true*’.

(NIGP2)

### 3.4. TDF Domain: Social/Professional Role and Identity

#### 3.4.1. Emergent Subtheme: Social Identity

Many HCPs acknowledged that being physically active (and being seen to be physically active) was a part of their social identity and that their perceived position of social influence and responsibility can be utilised to positively motivate patients to change behaviour.

#### 3.4.2. Emergent Subtheme: Professional Identity

A subtheme that was consistently discussed was that promoting physical activity in routine practice should be a part of every HCP’s job. Several HCPs identified that professional boundaries/roles (actual and perceived) may present a barrier to the application of physical activity to routine practice, and that there is potential for service development in some healthcare settings in addressing these barriers, so that all HCPs feel supported to apply physical activity to their routine patient care.

#### 3.4.3. Emergent Subtheme: Organisational Support for Physical Activity Promotion

Several themes emerged from interviews related to actual and potential organisational (structural) support(s) for the application of physical activity in routine practice. For example, multi-disciplinary team (MDT) working was valuable to applying physical activity to routine practice. This was a consistent theme across healthcare settings, sectors, and regions (see Table 2 and Supplementary Table S1). Furthermore, a ‘culture’ of promoting physical activity at a departmental/organisational level was important to ensuring the effective application of physical activity promotion to routine practice. Several HCPs highlighted that there was still scope for improvement in addressing physical activity per se in comparison to other health promotion areas addressed in routine practice.

### 3.5. Integrating Physical Activity in Routine Practice

The emergent domains and subthemes for integrating physical activity promotion in routine practice are outlined and discussed below under patient assessment, discussions with patients about physical activity, and prescribing physical activity.

### 3.6. Patient Assessment

HCPs were asked about assessing levels of physical activity with patients in routine practice.

### 3.7. TDF Domain: Skill

#### 3.7.1. Emergent Subtheme: Assessing Physical Activity as Part of Routine Practice

In general, assessing physical activity in routine practice, across all healthcare professions, followed an informal approach, relying on a conversation between HCP and patient to establish a patient’s levels of physical activity, with several HCPs identifying the adoption of a more ‘formal’ assessment as an area for (potential) CPD. Assessing physical activity as a ‘Vital Sign’ was discussed. More specifically, general practitioners (GPs) identified this as a concept that could contribute to the promotion of physical activity in their routine practice.

#### 3.7.2. Emergent Subtheme: Assessing Functional Status

Assessing functional status with patients was notably more structured, which may reflect the principle aims of restoring and management of functional status in falls prevention and preserving independence in activities of daily living that were discussed by many HCPs.

### 3.8. Discussions with Patients about Physical Activity

HCPs were asked how often they discussed physical activity with patients, how they initiated the conversation, and what were the barriers and facilitators to discussions about physical activity in routine practice.

### 3.9. TDF Domain: Memory, Attention, and Decision Processes

#### Emergent Subtheme: Models of Consultation

Several HCPs reported that they were guided by how the consultation unfolded, and the rapport that was developed with the patient during the consultation as to whether to discuss physical activity. Several discussed strategies to address common barriers that patients may put forward to not wanting to engage with physical activity and also discussed that routine promotion of physical activity was reinforced by positive patient feedback.

### 3.10. TDF Domain: Environmental Context and Resources

#### 3.10.1. Emergent Subtheme: Barriers to Physical Activity Promotion in Routine Practice

A number of individual level (patient), organisational (waiting lists/caseloads/staff resource), and societal (cultural role of physical activity) level barriers were identified across the health professions to the routine integration of physical activity promotion in discussions with patients (See Table 2). For example, a patient’s engagement (or perceived engagement) with physical activity promotion was identified as a potential barrier to the integration of physical activity in routine patient care.

A lack of time to routinely integrate physical activity in patient consultations, lengthy patient waiting lists, and large caseloads was a commonly cited theme. Limited resources (e.g., staffing levels) were also identified as barriers which impact the capacity of HCPs to effectively integrate physical activity promotion in routine care, and for older adults in particular, several HCPs identified that a barrier was the ‘cultural’ role that physical activity plays in the lives of many older adults on the island of Ireland (see Table 2).

#### 3.10.2. Emergent Subtheme: ‘Physical Activity’ or ‘Exercise’ as Part of Routine Care

There was a general lack of clarity and consistency with the terms used interchangeably, and several HCPs identified the need to differentiate between the terms ‘exercise’ and ‘physical activity’ with patients as ‘exercise’ has potential negative connotations for some older adults. Several HCPs identified a need to develop the context for its use in discussions with patients in routine care, and several HCPs identified a need to address the correct use of terminology as a potential area for further CPD. One participant highlighted a professional body scheme (physiotherapy) which was developed to address this,

‘*…there was a campaign over the last few years and it was about you know, ‘hate exercise, love activity’. That exercise is not just for the people who go to the gyms and take part in triathlons, but it can be incorporated into life*’.

(NIP2)

### 3.11. Prescribing Physical Activity

HCPs were asked about physical activity prescription, referral, and community practice linkage. As most interviews took place during the COVID-19 pandemic, participants were asked to reflect on ‘typical’ practice prior to the pandemic and reflect on their experiences of prescribing physical activity as part of routine practice.

### 3.12. TDF Domain: Environmental Context and Resources

#### 3.12.1. Emergent Subtheme: Exercise Is Medicine

The theme that ‘exercise is medicine’ arose in several interviews with HCPs, with the discussions typically involving the role that both the individual and the wider community can take in promoting physical activity to improve health.

#### 3.12.2. Emergent Subtheme: Practice-Based Resource

The MDT structure was highlighted as important for both integrating physical activity in routine care and for promoting community-practice linkage.

#### 3.12.3. Emergent Subtheme: Social Prescribing

The importance of having a ‘community navigator’, either within the structure of the MDT, as part of the wider integrated care partnership or embedded in the community was highlighted in several interviews as important for the integration of physical activity in routine care, with one HCP highlighting a networking opportunity as a potential mechanism to facilitate knowledge translation of service provision at a community level (see Supplementary Table S1).

#### 3.12.4. Emergent Subtheme: Community-Based Resource

The range and availability of community-based resources for HCPs to refer into was highlighted as a significant facilitator in integrating physical activity in routine care, whether that referral was to another community-based HCP, or to an established community-based programme run by a range of providers that had sufficient and appropriate structures in place to support patients. It was highlighted frequently that a well-developed and resourced program can have significant benefits for increasing participation in older adults, for example,

‘*We were told that older people wouldn’t come in for the exercise class. We were told they just don’t do it. And I think by the end of our last class, we would have run three classes over a week and had 45 patients in*’.

(RoIP15)

However, it was also clear that the range and availability of community-based resource(s) varied considerably between practices and communities, from state-of-the-art facilities to little or no access to onward referral.

### 3.13. Developing Practice to Support the Application and Integration of Physical Activity Promotion

Several areas for potential service development were highlighted that could support HCPs to integrate (or further integrate) physical activity into routine practice. HCPs’ quotations for emergent themes on developing practice to support the application and integration of physical activity promotion are presented in Table 3.

## 4. Discussion

Current evidence suggests that appropriate education, training, and access to resources are essential for supporting promotion of physical activity in routine practice for older adults [14]. This research sought to explore these themes in detail and adds HCPs’ own views on the supports that are needed to effectively apply and integrate physical activity promotion in routine practice. It explores these concepts across a wide range of healthcare professions, settings and sectors, and aligns emergent themes to key theoretical domains of HCPs’ behaviour to further our understanding of this area.

The key findings from this research confirm that focused education, appropriate training, and access to tailored resources are all essential to support the promotion of physical activity in routine practice. In addition, this research highlights that for these supports to be effective, a ‘cultural shift’ is required in HCP training and health service provision to adopt the routine application and integration of physical activity promotion in the health services.

### 4.1. Applying Physical Activity to Routine Practice

Many HCPs in this study highlighted continuing education and skill development as essential to raise their confidence and competence to undertake assessment and provide brief advice and/or counselling on physical activity in routine practice with older adults. Many accredited their ‘working’ knowledge of physical activity in prevention and treatment to continuing professional development rather than to their undergraduate/initial education and training. This is consistent with previous research that has highlighted the need for postgraduate training for HCPs to effectively address health behaviour change in routine care [20] and highlights the need for continuing support in the development and maintenance of programmes and interventions that facilitate HCPs continuing knowledge development.

### 4.2. TDF Domain: Knowledge

Four subthemes emerged under the TDF domain of ‘Knowledge’ (see Table 2). Discussions with HCPs consistently turned to the need for increased service provision of training and practice development to support knowledge development of the application of physical activity in routine practice with older adults. Increasing continuing professional ‘knowledge’ development is particularly important given that recent research has highlighted that HCPs’ knowledge of physical activity guidelines varies considerably across healthcare professions and that having a detailed knowledge and recall of physical activity guidelines was associated with formal assessment, initiating discussion, and referral/signposting to physical activity services as part of routine practice [14]. In this research many HCPs also identified that there was still scope for improvement in addressing physical activity in comparison to other health promotion areas addressed in routine practice with older adults. Previous research suggests that HCPs consider physical activity to be less important than other health promotion activities such as smoking cessation [21]. In this research HCPs identified ‘focused training’; ‘promoting available resources’; ‘education on behaviour change techniques’ and ‘displaying infographics’ as potential areas for service development to promote HCPs’ knowledge development of the application of physical activity to routine practice (See Table 2 and Table 3).

### 4.3. TDF Domains: Social/Professional Role and Identity

HCPs also highlighted that knowledge development impacts on a HCPs ‘professional identity’: the set of behaviours and displayed personal qualities of an individual in a work setting (TDF domain: Social/Professional role and identity) [17]. Several HCPs identified that professional boundaries/roles (actual and perceived) can affect their confidence to apply physical activity to routine practice. Indeed, a recent study among nursing students in Ireland highlighted a lack of confidence in physical activity and recommended the integration of more physical activity education into the nursing curriculum to equip the future nursing workforce with the skills and confidence they need to promote physical activity to their patients [22]. Several participants identified MDT working as a model of good practice that facilitates the application of physical activity to routine care of older adults by removing potential role-related barriers. It was suggested that integrated models of practice also helped to establish a ‘culture’ where every HCP works effectively together to ‘share’ knowledge and apply physical activity to a patient’s care.

Changing the ‘culture’ of health services to apply physical activity promotion as part of disease prevention requires leadership throughout organisational structures, and clinical leadership is essential in demonstrating support for the development of programmes and services that have the potential to reduce the burden of chronic disease [20]. The current Making Every Contact Count (MECC) program, to provide training in brief (and opportunistic) interventions to all healthcare professionals who may have patient contact in Ireland and Northern Ireland has highlighted the need for a long-term commitment to training support of HCPs in this role, and the need to reach a critical mass of trained staff to implement this agenda [20].

### 4.4. TDF Domain: Belief about Consequences

Several HCPs cited their belief in the benefits of physical activity for both older patients and their own health, and that continuing professional development in this respect was central to embedding physical activity in their routine practice. It also impacted on their ‘social identity’, with several of those interviewed (particularly GPs) highlighting that they were keen to been seen to ‘practice what they preach’. ‘Health and wellbeing programmes’ for HCPs was identified as an area for potential service development to foster knowledge and subsequent belief of the benefits of physical activity that may transition into routine practice. The national physical activity plan for Ireland highlights the pivotal role that HCPs play in promoting physical activity and that they should be supported to lead more active lives through supportive workplace practices and policies [23]. Indeed, research suggests that clinicians who are physically active themselves are more likely to counsel patients about physical activity in routine care and may serve as a more convincing role model to their patients [24].

### 4.5. Integrating Physical Activity Promotion in Routine Practice

In addition to applying physical activity to routine practice, HCPs were asked about integrating physical activity promotion into routine practice. The emergent domains and subthemes for integrating physical activity promotion in routine practice are outlined and discussed under ‘patient assessment’, ‘discussions with patients about physical activity’ and ‘prescribing physical activity’.

### 4.6. Patient Assessment: TDF Domain: Skills

The assessment of physical activity levels by HCPs in routine practice is the cornerstone of the counselling process [24] and is a key action recommended by the WHO to promote health-enhancing physical activity. In this study, ‘formal’ assessment of physical activity as part of routine practice was identified as an area of potential service development across all health professions, but particularly in general practice where it could be supported through integration in electronic medical records (EMR)/IT systems. Embedding physical activity as a ‘vital sign’ in EMR with ‘pop-ups’ to prompt the assessment of physical activity is both feasible and effective but requires training, appropriate infrastructure, and incentive to effectively integrate into models of patient consultation [24].

Several barriers were identified by HCPs in integrating physical activity assessment in routine practice with older adults, including ‘time’ and ‘lack of incentive’. These barriers are reported widely within the literature [25]. Previous research has also highlighted that the lack of formal assessment in routine practice may reflect the level of training and support that HCPs have received on physical activity promotion broadly, and on physical assessment more specifically [14]. Nonetheless, many HCPs in this study identified that physical activity assessment should be standard practice in every patient consultation due to the numerous physical and mental health benefits for patients. It was felt that such future programmes and interventions that promote physical activity in the health services should contain appropriate and standardised training on physical activity assessment as part of routine care.

### 4.7. Discussions with Patients: TDF Domain: Memory, Attention, and Decision Processes

HCPs acknowledged that they are widely respected and trusted, and as such they have considerable potential to influence public and individual opinion, but they also reported that they face challenges in discussing physical activity with patients, namely ‘time pressures’ and ‘caseloads’. HCPs also reported that their perception of an older patient’s motivation to receive advice or discuss physical activity was an important component in decision making as to whether to initiate a discussion about physical activity. ‘Motivational interview training’ in the emergent subtheme ‘Models of consultation’ (TDF domain: Memory, attention, and decision processes) was highlighted as a potential area for practice development to support HCPs to anticipate barriers (such as patient motivation) and discuss patient-centred solutions to effectively address these barriers. Further to this, ‘Education on behaviour change techniques’ was also identified as a necessary component of initial and continuing professional education (TDF Domain: Knowledge). Evidence demonstrates the increased effect of brief physical activity interventions that use valid (and multiple) behaviour change methods, namely, behavioural, cognitive, and motivational approaches [7].

### 4.8. TDF Domain: Environmental Context and Resources

HCPs also highlighted several ‘organisational’ and ‘societal’ barriers to the routine integration of physical activity in discussions with patients, and this was consistent across professions and healthcare settings. Several participants identified that greater ‘Investing in prevention’ was required to adequately fund training and resources to support HCPs to effectively integrate physical activity promotion into routine practice and to move away from a ‘treatment’ model of healthcare to ‘prevention’, but to support this, campaigns are required to raise both HCPs and the general public’s awareness of the benefits of physical activity through ‘Supportive public health campaigns’. Public education campaigns that involve mass, digital and social media, outdoor billboards and posters, and mass distribution of information are an effective way to transmit consistent and clear messages about physical activity to a large population, and have been highlighted as one of eight investments that ‘work’ for physical activity in a call to action for embedding physical activity in national and subnational policies [10].

### 4.9. Prescribing Physical Activity: TDF Domain: Environmental Context and Resources

Prescribing ‘exercise as medicine’ and signposting older adult patients to physical activity services (i.e., exercise referral programmes/community-based physical activity initiatives) in routine practice has been shown to be associated with a detailed knowledge of the application of physical activity to routine care [14]. How the topic is raised and linked to a patient’s specific health conditions is central to patient acceptance to the topic [13]. Research also suggests that the ‘motivation’ provided by HCPs is key to whether a patient accepts offers of being signposted to community physical activity opportunities [26,27], highlighting again the need for specific and ongoing education and training on exercise prescription as part of routine care. In this study, several participants discussed models of good practice in implementing referral pathways from both primary and acute care to community- or university-based programmes. They described how these models can provide that ‘motivation’ to patients through continuing care and support, tailored to their needs, in a local community setting with programmes that provide the key techniques for behaviour change (e.g., goal setting and social support) that are essential to the adoption and maintenance of a more physically active lifestyle [28].

It was clear in interviews with HCPs, however, that these models of referral were ‘pockets’ of good practice and, in general, there was significant variability in access to and availability of community-based resources across settings and professions. Previous research on the requirements for community-based provision has consistently highlighted the need for better community-based collaborations with sport, leisure, and fitness providers, but also improvements in infrastructure to support physical activity behaviour change [25]. In this study, in-service support through resource allocation and funding was highlighted as necessary in ‘supporting the development of innovative physical activity programmes’ whereas those HCPS who had shown initiative and developed a service to link community and practice have received retrospective health system funding to maintain that service (see Table 2).

Multi-disciplinary team working also facilitated patient referral utilising the available HCP resource within a practice. HCPs who worked in a setting that had access to a social prescriber also identified this as an extremely valuable resource, highlighting that this individual or service was key to the development and maintenance of effective community-practice linkage through ‘community resource mapping’ (see Table 3). A framework for the integration and mainstreaming of social prescribing within the health service in Ireland has recently been published [29].

### 4.10. Strengths and Limitations

This study captured views from a diverse range of healthcare professions and the TDF was utilised as an evidence-based method for identifying determinants of HCPs’ application and integration of physical activity promotion in routine practice. It involved 63 interviews across two jurisdictions and two different healthcare systems. However, consideration should still be given to the generalisability of the study findings. Selection bias is an issue that needs to be considered in this context, as it is possible that HCPs who are interested in and utilise physical activity in routine practice were more motivated to participate. The smaller number of respondents from general practice and nursing (relative to physiotherapy and occupational therapy), and the predominately female sample is also a potential limitation of the research. However, the overall patterns of participation in this study are consistent with other studies conducted in this area and reflect the gender profile of the four professions.

## 5. Conclusions

Irrespective of profession, focused education, appropriate training, and access to tailored resources are all essential to support the promotion of physical activity in routine practice. However, it is evident that for such supports to be effective, a ‘cultural shift’ is required in HCP training and health service provision to adopt the growing evidence base that physical activity promotion must be part of disease prevention and treatment in routine practice.

Support programmes and campaigns to develop wider societal knowledge of the role of physical activity in health must be part of this process. There needs to be a shift in age-based assumptions around physical activity that challenge both the HCP and the older person themselves about what is possible and beneficial as we grow older. However, at the core is the need for service and practice development to support the routine application and integration of physical activity promotion in the health services.

Further research is required to explore the feasibility of implementing the recommendations by HCPs on the application and integration of physical activity promotion in routine practice.

## Figures and Tables

**Table 1:** Characteristics of interview participants.

	All Interviewees	
	N	%
Professional affiliation		
General practitioner	10	15.87
Physiotherapist	24	38.10
Occupational therapist	19	30.16
Nurse	10	15.87
Gender		
Male	10	15.87
Female	53	84.13
Years qualified		
0–5	2	3.17
6–10	10	15.87
11–15	12	19.05
16–20	20	31.75
21–25	8	12.70
26+	11	17.46
Healthcare setting		
Primary	44	69.84
Secondary	5	7.94
Other (e.g., residential)	14	22.22
Health sector		
Public	55	87.30
Private	7	11.11
Other	1	1.59
Region		
Northern Ireland	22	34.92
Ireland	41	65.08
Total	63	100

**Table 2:** Healthcare professionals’ quotations for emergent domains and subthemes on application and integration (assessment/discussion/prescription) of physical activity in routine practice.

**Applying physical activity to routine practice**	**TDF Domain**	**Subtheme**	**Exemplar Quotation**	**Area(s) Identified by HCPs for Potential Service Development**
	**Knowledge**	Understanding health benefits	*‘There’s good evidence that exercise, the strength and balance programmes, can significantly reduce falls, so there’s a big payback there, in terms of preventing hip fractures’ (NIGP1).*	**Focused training** *‘… with my nursing background and my health promotion practice, they’re really important messages for us to be sharing with the frontline staff, and then we have very strong evidence around the benefits of promoting health-enhancing physical activity to keep people active as they age, which will help to reduce their unhealthy weights and also reduce the risk of chronic conditions, or maybe delay the onset of chronic conditions…’ (RoIN3).*
		Source(s) of knowledge development	*‘I keep myself updated through the Public Health Agency, you know, anything that they would promote and publicise. And I try to link in with Councils and with my Health and Wellbeing Team, whenever it comes to them giving health promotion advice’ (NIN2).*	**Promoting (available) resources** *‘So, I think obviously though, there is a lot out there that we can, you know, the use of apps and the NHS website now even, there’s a lot of stuff to promote healthy lifestyle and there’s a lot of free resources there. So, I am aware of those but I’m sure there’s plenty I’m not aware of as well’ (NIOT5).*
		Initial and continuing professional development	*‘… since I qualified many years ago, all of my knowledge around guidelines, around activity would have been since graduation and it would have been from those courses’ (NIP2).*	**Education on behaviour change techniques** *‘I think we need to be… that whole psychology of it needs to be strengthened and developed more and that buying in and how we sell it…’(RoIP3).*
		Knowledge of physical activity guidelines	*‘I know the guidelines are there, am I up to date on them? Probably not but yeah, I obviously understand the importance of physical activity and make sure that that is brought into all treatments where feasible’ (NIP1).*	**Displaying Infographics** *‘And as well then the government guidelines for physical activity, I wasn’t totally aware of the exact recommendations of it until about two or three years ago. And those infographics now are everywhere in our department and everyone’s very aware of the importance of physical activity, probably more so since then’ (NIP4).*
	**Belief about consequences**	Belief of the benefits for health	*‘…but I suppose we believe, I think that’s the thing, I have no doubt of the benefits of exercise’ (NIGP2).*	
	**Social Professional Role and Identity**	Social identity	*‘There is role modelling with it which I am quite keen to promote. Again, sometimes at lunch time I will go out running and I am quite keen that people recognise me as somebody who does that’ (NIGP3).*	**Health and wellbeing programmes for HCPs** *‘I think there needs to be a bigger emphasis on health and wellbeing for staff before you’ll probably see a huge knock-on effect for patients, or maybe you know concurrently even’ (RoIP14).*
		Professional identity	*‘And how important it is. And I think like when you’re, yeah, it needs to be everyone’s role definitely’ (RoIOT3).* *‘Well I think it’s everyone’s realm‘(NIP4).*	**Reinforcing good practice that supports every HCP to promote physical activity** *‘We would do in-service together and you know, have team meetings and we’re always sort of asking what are you doing about this and how you’re using this, what leaflets are you using, things like that. What outcome measures are you using? And like we all, in different areas had the falls prevention class and we were using that really well and everybody seemed to be enjoying it. So, from that point of view you know, we all seem to be doing the same thing’ (NIP5).*
		Organisational support for physical activity promotion	*‘So, we would have speech and language, we would have a dietician, we’ve nursing staff, physiotherapists, myself, OT and then once a week we have a geriatrician here as well which is brilliant, and then we have an SHO who’s here every day as well. So, we’re lucky you know, we’re in a very good environment and we’re very open and we’re always open to trying different things. Like we had a wellness group here. We’ve done yoga before’ (RoIOT2).*	**Identifying supports for service delivery** *So, in some of the areas we had a follow-on programme of 16 weeks, that was delivered again by [council service provider]. But not all areas within our trust had that. So, the Public Health Agency after I had brought that up, they fund the 16-week programme for all areas. I also said about the lack of weights, to be able to get the progressiveness with their exercises. And the Public Health Agency funded the weights (NIP6).*
**Integrating physical activity promotion in routine practice: Assessment**	**Skills**	Assessing physical activity	*‘But I always say, ‘What are your physical activity levels at the minute? What are your physical activity levels? What do you do?’ They always say, ‘I walk or play golf.’ I always add it into the initial assessment or the chat with them’ (NIP4).*	**Formal assessment of physical activity** *‘Yeah, I suppose we don’t, in our team we don’t really have a structure. It’s something that probably needs to be put in place. Obviously in our assessment there will be, you know, previous mobility, it’s basically as far as it goes’ (NIP1).*
		Assessing functional status	*So, generally, we do a comprehensive balance assessment initially. So, even if they’re coming in with back pain, we still do a balance assessment with them to see where they are. We’d also do a bone health assessment with them. So, we obviously look at their bone health, particularly if they’ve had a recent fracture’ (RoIP15).*	
**Integrating physical activity promotion in routine practice:** **Discussion**	**Environmental context and resources**	Barriers to physical activity promotion	Individual level (e.g., perception of patient motivation)	**Motivational interview training (see below) and tailored support** *‘The barriers more are where people who say they physically can’t do it. They say, ‘Oh, I have got a bad heart and I have bad knees.’ There is always some excuse, but we tailor the programmes to meet those needs as well and try to educate them a wee bit more in that they still can do light exercise and what are the health benefits for that. Most of them do try and do some sort of activities’ (NIOT4).*
			*‘And I’m trying to think… yeah, we had another man who went home, but he was much, much less motivated, so that conversation would have been much shorter’ (RoIP7).*	
			Organisational (e.g., time)	**Investing in prevention; Incentivising the use of physical activity in routine practice; Training and Practice Development and Service provision** (see Table 3)
			*‘I think that’s always been a big problem with GPs, is the lack of time you have with people for the lifestyle counselling and things’ (NIGP5).*	
			Societal (e.g., culture of physical activity)	**Supportive Public Health Campaigns** (see Table 3)
			*‘I just think ageing in Ireland has a different concept of itself, I don’t think like, like I have obviously been on webinars where we’ve had Australians speaking and they talk about the older people over there having FitBits and going out walking, they’re recording their steps and they’re being very proactive about their exercise. Whereas I don’t think that has filtered into Ireland or into the culture here yet’ (RoIP5).*	
		‘Physical activity’ or ‘Exercise’ as part of routine care	*‘I think people’s eyes glaze over when the word exercise is mentioned. In a lot of places, it’s actually not a very motivating word. So, I think certainly, physical activity, or broadening the scope of it, and the use of language, will help with motivating people’ (RoIP2).*	**Focused Training in Clinical linguistics** *‘At our [Professional body] conference this year, there was a guy giving a presentation on clinical linguistics, and the focus was on the language around pain. But a lot of the guidelines that he gave us would very much apply to motivating people in relation to physical activity. So, I think we probably have a lot of work to do still’ (RoIP1).*
	**Memory, attention, and decision processes**	Models of consultation	*‘So, yes, I would say the vast majority of it is off my own experience and just probably reading the situation and learning from what I’ve done before in the past. So, yeah, yeah, no I can’t really say I have formal teaching in that’ (NIGP4).*	**Motivational interview training** *‘I would informally to myself go even with that approach and sort of chatting through on what’s the person’s goal and non-confrontational and rolling with resistance and all of that, even with that if the person is evidencing, they’re just not interested’ (RoIP7).*
**Integrating physical activity promotion in routine practice: Prescription**	**Environmental context and resources**	Exercise is medicine	*‘But really, the more you hear about it, the more it’s usually beneficial in management of so many conditions and the prevention of so many conditions’ (NIGP4).*	**Physical activity integrated into IT systems** *So, again, in our own software, it is individually, if I am putting in a blood pressure, I type in blood pressure and then temperature is a separate thing again. So, there isn’t—I would have to look at exercise there. So, I don’t think there is any formal exercise dialogue box there, if you like, as far as I know’ (RoIGP5).*
		Practice-based resource	*‘We also have a practice physio, but that would be more for people with specific orthopaedic conditions, back or knee pain, where you’re sort of recommending specific exercises for that particular problem’ (NIGP2).*	**Supporting the development of innovative physical activity programmes** *‘So, then we trained up our own staff and we did like a walking group, we got that started and then we could pass over to the likes of [service provider]. We just kind of formed that bridge, tried to anyway in terms of physical activity’ (NIP8).*
		Social prescribing	*‘But there was social prescribing, you know, where you could refer to a local counsellor essentially, a local agent I suppose who knew what services were available in the area and can signpost people to what they needed’ (NIGP4).*	**Community resource mapping** *‘The biggest issue is that people know of pockets of good practice and people don’t know what the landscape is so people don’t know, we have no register or geographic map of what supports are out there or where you might tap into exercise’ (RoIP10).*
		Community-based resource	*‘[Name of Charity] have a tremendous exercise programme that we can refer patients as a referral, but patients can also contact themselves, which is walking clubs and gardening clubs and those sort of more normal physical activity, physical activity, but done in groups that stimulate people to make friendships and to keep it up more long-term’ (NIGP1).*	**Promoting community-practice linkage** (see Table 3)

TDF: Theoretical Domains Framework; NIGP: Northern Ireland-based general practitioner; RoIGP: Ireland-based general practitioner; NIN: Northern Ireland-based nurse; RoIN: Ireland-based nurse; NIP: Northern Ireland-based physiotherapist; RoIP: Ireland-based physiotherapist; NIOT: Northern Ireland-based occupational therapist; RoIOT: Ireland-based occupational therapist.

**Table 3:** Healthcare professionals’ quotations for emergent themes on developing practice to support the application and integration of physical activity promotion.

Area of Support Identified	Exemplar Quotation
Investing in prevention	*‘And so, if part of our role is to preserve life and the easiest way to do that and one of the cheapest ways is by promoting a healthy lifestyle… So, it would be in the government’s interest and in medical schools’ interest and things like that to be putting funding and resources into those, into raising awareness’ (RoIGP4).*
Incentivising physical activity in routine practice	*‘I think money is just, I’m simplifying it, but I think that if you want to get, you know if the planners are saying, you know, moving, being active is going to be good for your health, it’s going to be saving money in the long… it’s going to be good for people’s health, which is the most important, but it’s going to save money on hospitals, on medications down the line, well then we should invest in it, and if we’re going to task professionals with promoting it, we should pay them’ (RoIGP2).*
Promoting community-practice linkage	*In general practice…* *‘Put the resources in a practice, like the MDT scheme, that helps them, that enables them. If you have a local resource, get them to come out to your practice and talk to you about it, rather than dumping them with a big bundle of papers’ (NIGP3).*
	*In the acute setting…* *‘… like it would be great to have the knowledge about everything that’s available in your catchment. But the reality is that that doesn’t really happen, and a lot happens in the community that in the acute setting you’re not aware of. Likewise, things happen in the acute setting that the community aren’t aware of, I think it needs to be a bit broader than, you know, just limited to your own environment’ (RoIN7).*
Training development	*In general practice (focused training)* *‘… practical educational sessions that would be tailored to general practice, that would be based on the consultation. So, it would be a kind of a simulated workshop based on GP consultations, where you’re basically demonstrating how this is done. A case-based simulation—active one-hour session that would be based locally for GPs and where GPs would be rewarded for going by getting CPD. Ultra-focused. It’s not trying to do everything, and maybe the GP gets one skill and one practice-based tool out of it and no more’ (RoIGP2).*
	*In Nursing (training and resource development in residential care)* *‘… but some education, very practical, quick, easy, instructional maybe, multimedia, videos, laminated cards, about how we can introduce physical activity into everyday activities would be really, really helpful and I think it would be really beneficial to residents’ physical and mental health’ (RoIN6).*
	*In Physiotherapy…* *‘… good governance and clear training and ongoing CPD and you know, it’s a challenging area. But I think it’s a really needed area. And I think if you’re looking to really you know, the gold standard and really improve, this is one really nice way of doing it’ (RoIP3).*
	*For support staff (healthcare assistants)* *‘I think even general staff like healthcare assistants, particularly on a rehab ward. I think there should be some type of training for them, and I know there’s staff pressures and stuff, but I think probably education would be a big thing and training for unqualified staff, to support with that gap in-between therapy’ (NIOT5).*
Practice development	*‘Yeah, so we need the service, so the health services to introduce physical activity competency as, I suppose, a quality indicator or an area, a specific area of work in health professionals’ assessment in treatment of staff. So, it needs to be very explicit in terms of, you know, it being a core component of patient interventions, but we also need the professional bodies to actually, I suppose include it as a competency in terms of professional practice’ (RoIN3).*
	*‘And I think if your standards and your compliance was measured those standards and that included how you integrate physical activity into the daily care that you deliver, and how you report on that in terms of your nursing documentation and your record keeping, I think that would go a long way to making sure that it became part and parcel of what we do’ (RoIN6).*
Service provision	*‘I think unless there is more education, it will probably be like a status quo. I think it takes an education programme as to the importance of mobility. But unless they improve the staffing levels and improve the education, I think they could quite quickly be neglected’ (NIN1).*
Physical activity awareness campaign for staff (and public)	*Supportive public health campaigns* *‘… so you’ve got the public health champions and, you know, people, well-known sports stars promoting it and then you’ve got GPs on as well giving that message so that what the GP is doing is part of a greater movement for the good, and the GP is tying into it and it’s natural and it feels easy and good and right to tie into that’ (RoIGP2).*

NIGP: Northern Ireland-based general practitioner; RoIGP: Ireland-based general practitioner; NIN: Northern Ireland- based nurse; RoIN: Ireland-based nurse; RoIP: Ireland-based physiotherapist; NIOT: Northern Ireland-based occupational therapist.

## Data Availability

Data is contained within the article or Supplementary Material Table S1.

## Supplementary Materials

The following are available online at https://www.mdpi.com/article/10.3390/ijerph182111222/s1, Table S1: Additional healthcare professionals’ quotations for emergent domains and subthemes on the application and integration (assessment/discussion/prescription) of physical activity in routine practice.
