# Supplementary material for: Healthcare Professionals’ Application and Integration of Physical Activity in Routine Practice with Older Adults: A Qualitative Study
Source: Int J Environ Res Public Health. 2021 Oct 26;18(21):11222. doi: 10.3390/ijerph182111222 (PMC8582732; doi:10.3390/ijerph182111222)
Supplement: Supplementary file 1 [file ijerph-18-11222-s001.zip › ijerph-1391054-supplementary.pdf]

Table S1: Additional Healthcare professional's quotations for emergent domains and sub-themes on the application and integration (assessment/ discussion/ prescription) of physical activity in routine practice

	TDF domain	Sub-theme	Exemplar quotation
**Applying physical activity to routine practice**	**Knowledge**	Understanding of the benefits of physical activity for patient's health	*'And there's increasing evidence of the benefit. And particularly, the people that we see that tend to have those problems – obesity, diabetes, hypertension and mental health issues. I think the widespread strength and balance programmes are particularly important for the elderly and frail, or people who are getting towards that group' (NIGP1).*<br><br>*'The importance of mobility to elderly people, even from a dignity point of view, from a mental health point of view. Being able - the more mobile they are, it improves their whole wellbeing as well as mobility. And the importance of it couldn't be emphasised enough. And I think there should be more emphasis on maintaining mobility and improving mobility of all patients and residents' (NIN1).*
		Source(s) of knowledge development	*'Maybe just a few people that I follow (on Twitter) or maybe have an interest in physical activity and then also like the British Journal of Sports Medicine and that. I find their infographics really useful, so I tend to use that' (NIP9).*<br><br>*'I've been to a number of CME (Continuing Medical Education) meetings where this has been talked about and I just, I see examples of it. You know you can, here at work, I see examples of how lack of exercise can harm you' (RoIGP1).*
		Initial and continuing professional development	*'Yeah, unfortunately there wasn't actually a whole lot to be honest with you. I distinctly remember doing a few lectures or professional competency sessions on health and exercise. Were kind of on really detailed calorie counting. Complex equations that I've never used since, unfortunately. Very minimal practical applications' (RoIGP4).*<br><br>*'No, no, that's just myself and it's literally I've done a couple of, at this stage online modules. So, it's something that is I mean, it's offered in the trust and like it wouldn't be undergrad at all, it's postgrad, well it's not undergrad as far as I'm aware' (NIP8),*

	Knowledge of physical activity guidelines	*'I know a wee bit, but I wouldn't proclaim to be an expert at all. I would be aware of the importance of exercise and the importance of prevention of muscle wasting and bone density, the improvement of bone density. I would be aware of that now, but the exact guidelines on it, I wouldn't be able to quote you on them' (NIN1).*<br><br>*'So, I think it's really important as part of being a physiotherapist to make sure people are aware of the guidelines and to know how they can work towards achieving them and why they should be. So, I think it's, yeah, that is part of kind of routine practice in terms of asking how active someone is but in terms of my own personal practice I try to be kind of specific in terms of how many minutes and in what way' (RoIP12).*
**Belief about consequences**	Belief of the benefits of physical activity for health	*'I can only speak from my own cohort of patients unfortunately; they're either being diagnosed with cancer or they're following on from cancer treatment. I think they have a huge burden on their shoulders, I'm a firm believer that, walking and concentrating on your breathing, take your mind off your diagnosis and it can help you to focus a little bit away from you know, the negative impact of your diagnosis and your treatment' (RoIN7).*
**Social Professional Role and Identity**	Social identity	*'I think there's no point saying something if you're not doing it yourself, and I think it's clear that I do it because, well anybody who looks sees that I go for a walk at lunchtime. I do not look at that computer at lunchtime. I go off. I don't do lunchtime meetings. I'm out walking along the prom' (RoIGP1).*<br><br>*'I think they look to the GP as a source of expert advice on their health in general and I think we're privileged that a lot of patients would kind of trust our guidance and our opinion. And so, yeah, I think that a GP is ideally placed for this' (RoIGP4).*
	Professional identity	*'it's everybody's role and I think we need to try and bring it down to the lowest common denominator where we're encouraging people to move at all stages of their day and in all their activities' (RoIN6).*<br><br>*'I suppose in my roles there has always been a link at part of the multidisciplinary team with physiotherapy. And maybe there's been a bit of role segregation where nurses have traditionally left the kind of physical activity, physiotherapy side of things to physios. And as a result, I don't think it's been sort of mainstreamed into nursing care'(RoIN6)*

			'but purely from an OT educational side I suppose, I'm not involved with what the physio is doing and saying (RoIOT3).
		Organisational support for physical activity promotion	*'It's very easy to link in. We'd have joint sessions quite often with the Occupational Therapist. As I say, the MDT meetings there's a representative from each discipline at the meeting and if there was something that came up beforehand, you'd just go and you'd link in. And then we would have a communication system with the Nurses' (RoIP7*).<br><br>*'I'd certainly say that the message we're best at promoting is around smoking cessation. So, we've got this pretty solid evidence that GPs are willing to give brief interventions on smoking cessation and that it works. Then following that alcohol is probably number two. I think physical activity currently is well down the list' (RoIGP2).*
**Integrating physical activity promotion in routine practice: Assessment**	**Skills**	Assessing physical activity	*'There's probably not a formal structure here for assessing, probably, maybe there would be a falls assessment but again that's probably quite a low level' (NIP2).*<br><br>*'Yeah, I would always in terms of having that verbal conversation, I would always find out in terms of what their baseline would be, what their usual routine would be. Would they get themselves out for walks and what that walk would actually look like?' (RoIN4).*<br><br>*'It is kind of just conversation. I would consider it a blind spot really for me. It would kind of be maybe a known unknown. That I know that there must be assessment tools out there, but I just have never really incorporated them' (RoIGP4).*<br><br>*'Yeah, I suppose we don't, in our team we don't really have a structure. It's something that probably needs to be put in place. Obviously in our assessment there will be, you know, previous mobility, it's basically as far as it goes' (NIP1).*<br><br>*'No, because within the context of general practice, you don't have that much time to you know, use tools and things, you know, there are lots of physical activity questionnaires and things, but they can be quite time consuming and formulaic (NIGP5).*

			'I think it would be useful and clearly it would have you know, particularly it gets GPs thinking, you know, about what we're dealing with if you're dealing with that on a regular basis and you're starting to think well that's… You're maybe not doing it as much, whenever we actually talk about this. It's a very simple factor' (NIGP4).
		Assessing functional status	*'Well, we use the Barthel index. So, it's an outcome measure that looks at what somebody was at, at their baseline. It looks at all their ADLs (Activities of Daily Living), like washing and dressing and toileting, transfers of mobility. So, that is scored out of 20. Then you score it up on your initial assessment. So, it would determine whether somebody is off their baseline or not' (NIOT3***).***
	**Memory, attention, and decision processes**	Models of consultation	*'And so, giving them some kind of accountability but also motivation. Always being encouraging and using positive reinforcement. Even when they start to slip, you can talk about it being a bit of a blip and we'll get back on track now.' (RoIGP4).*<br><br>*..I said do you do much exercise, and he said, doctor, you told me five years ago to go for a walk every day, or two years ago, and since I followed your advice, I go out and walk ..every day, rain or shine. So, there are some patients who follow our advice' (NIGP2).*
**Integrating physical activity promotion in routine practice: Discussion**	**Environmental context and resources**	Barriers to physical activity promotion	*'I suppose in terms of physical activity, I mention it now and then and the reason for that is probably, I don't mention it on every contact.* ***Okay (Interviewer).***<br>*And the reason for that is probably sometimes maybe on my own part I can see, well sometimes I presume that they're not going to be like motivated because of how they are with me' (RoIOT3).*<br><br>*'I think we're competing with other priorities. So, I think actually at this stage the data and the evidence is ready, but our frontline practitioners are hugely busy and they have very big caseloads' (RoIN3).*<br><br>*'I think that's always been a big problem with GPs, is the lack of time you have with people for the lifestyle counselling and things' (NIGP5).*<br><br>*'..in an ideal world it'd be great to be able to cover it with every client but I think we just get side tracked and then you know, you're thinking about doing it and then you can only do I suppose what traditionally is your role. You think I'll go in and sort them pieces, purely from a pressure and staffing point of view' (RoIOT3).*

			'And of course, it's in the background of a society where we are much more sedentary, there's much more transport, the car is king. So, you do have to make an effort to counteract that' (NIGP2).
		'Physical activity' or 'Exercise' as part of routine care	*'rather than highlight the health risks… if you say listen you know, you'll feel better about this, you'll lose weight and you'll have more energy about you and you know, you will feel much (NIGP4)*<br><br>*'Where if I just say, it is good for their health, I don't think they take it on board. I try to build on it from an independence point of view saying, 'If I can keep you strong and, on your feet, you will be able to remain independent for an awful lot longer' (NIN2)*
**Integrating physical activity promotion in routine practice: Prescription**	**Environmental context and resources**	Exercise is medicine	*'I think the onus needs to be put back on people minding their physical health rather than there's a pill for every ill because unfortunately there isn't' (RoIP15)*
		Practice based resource	*'So, within the practice we have a physiotherapist, and then we've got even, you know, a mental health worker. They will both be recommending exercise' … And then we've got a practice based social worker who is key to all of it because she deals with the whole range of patients, but she's very much involved with kind of social prescribing and getting people aware of the resources and things that are available in the local area. (NIGP5).*
		Social prescribing	*'we are quite fortunate that we have access to social prescribing in our area. So, if there's somebody that's interested, I would talk about the option of a social prescriber, and in our area, we've set up local walking groups. So, I would mention that if I thought the person might be interested' (NIGP2).*<br><br>*'I think one of our [TEAM] ran a networking event where she had everyone kind of from local kind of exercise groups and local services. So that was a really nice way of us all linking in with each other and she came out with a really nice book of resources and advice' (RoIP12).*

	Community based resource	*'Well, I suppose we work individually mainly but if we needed – if our patients needed guidance as regards physical activity, if they are recuperating or coming out of hospital, we would then link in with the OT and the physios' (RoIN2).*<br><br>*'Yeah, so we would refer into the [charity] and they do a physical activity group and sort of a, a lecture as such, they do an hour of physical activity once a week with stroke patients' (NIP1).*<br><br>*'We were told that older people wouldn't come in for the exercise class. We were told they just don't do it. And I think by the end of our last class, we would have ran three classes over a week and had 45 patients in' (RoIP15).*<br><br>*'so we have a specific physiotherapy gym with parallel bars, a motorised exercise bike, weights, exercise bands, stepping equipment, so we can bring people into the gym who are able to do higher level exercises' (RoIP12).*<br><br>*'But like that, there aren't any weight management hubs or anything set up or anything. Like, it's all very itty-bitty' (RoIP5).*
